# Early Stage Markers of Late Delayed Neurocognitive Decline Using Diffusion Kurtosis Imaging of Temporal Lobe in Nasopharyngeal Carcinoma Patients

**DOI:** 10.7150/jca.48759

**Published:** 2020-08-25

**Authors:** Gang Wu, Shi-shi Luo, Priya S Balasubramanian, Gan-mian Dai, Rui-rui Li, Wei-yuan Huang, Feng Chen

**Affiliations:** 1 Department of Radiation Oncology, Hainan General Hospital (Hainan Affiliated Hospital of Hainan Medical University), Haikou, China.; 2 Department of Radiology, Hainan General Hospital (Hainan Affiliated Hospital of Hainan Medical University), Haikou, China.; 3 Department of Electrical and Computer Engineering, Cornell University, Ithaca, NY, USA; 4 Department of Radiology, Weill Cornell Medical College, New York, NY, USA.

**Keywords:** Diffusion kurtosis imaging, neurocognitive function, temporal lobe, nasopharyngeal carcinoma, intensity-modulated radiotherapy

## Abstract

**Purpose**: To determine whether the early assessment of temporal lobe microstructural changes using diffusion kurtosis imaging (DKI) can predict late delayed neurocognitive decline after radiotherapy in nasopharyngeal carcinoma (NPC) patients.

**Methods and Materials**: Fifty-four NPC patients undergoing intensity-modulated radiotherapy (IMRT) participated in a prospective DKI magnetic resonance (MR) imaging study. MR imaging was acquired prior to IMRT (-0), 1 month (-1), and 3 (-3) months after IMRT. Kurtosis (Kmean, Kax, Krad) and Diffusivity (Dmean, Dax, Drad) variables in the temporal lobe gray and white matter were computed. Neurocognitive function tests (MoCA) were administered pre-radiotherapy and at 2 years post-IMRT follow-up. All the patients were divided into neurocognitive function decline (NFD group) and neurocognitive function non-decline groups (NFND group) according to whether the MoCA score declined ≥3 2 years after IMRT. All the DKI metrics were compared between the two groups, and the best imaging marker was chosen for predicting a late delayed neurocognitive decline.

**Results**: Kurtosis (Kmean-1, Kmean-3, Kax-1, Kax-3, Krad-1, and Krad-3) and Diffusivity (Dmean-1 and Dmean-3) of white matter were significantly different between the two groups (*p*<0.05). Axial Kurtosis (Kax-1, Kax-3) of gray matter was significantly different between the two groups (*p*<0.05). By receiver operating characteristic (ROC) curves, Kmean-1 of white matter performed best in predicting of MoCA scores delayed decline (*p*<0.05). The radiation dose was also significantly different between NFD and NFND group (*p*=0.031).

**Conclusions**: Temporal lobe white matter is more vulnerable to microstructural changes and injury following IMRT in NPC. Metrics derived from DKI should be considered as imaging markers for predicting a late delayed neurocognitive decline. Both temporal lobe white and gray matter show microstructural changes detectable by DKI. The Kmean early after radiotherapy has the best prediction performance.

## Introduction

Radical radiotherapy is the preferred standard treatment for nasopharyngeal carcinoma (NPC)^1^. A limitation of targeted radiotherapy is damage to adjacent tissue, in the case of NPC, the temporal lobe is inevitably influenced by the radiation field^2^. The utilization of intensity-modulated radiotherapy (IMRT) greatly reduced the incidence of radiation-induced temporal lobe necrosis due to precision of radiation delivery^3^. Kam et al.^4^ revealed that IMRT was able to reduce Dmax to the temporal lobes in T4N2M0 NPC patients by 20.5 Gy compared with conventional RT. However, Hsiao KY et al.^5^ reported that 76.7% of patients treated with IMRT still had significantly lower cognitive functioning scores compared with their pre-radiotherapy scores.

Radiation-induced temporal lobe injury is categorized as acute (few days to few weeks), early delayed (1-6 months), and late delayed (6 months to few years)^6^. Radiation-induced temporal lobe injury can cause different degrees of neurocognitive function impairment through acute, early, and late delay radiation phase, and the clinical manifestations range from neurocognitive impairment to neurobehavioral impairment symptoms. Patients may recover from acute and early delayed effects, however late delayed injury presents as severe functional or cognitive decline following radiotherapy that is usually permanent^7^, ^8^. As such, it is of interest to track this late delayed response, as this will have a greater impact on patient prognosis in the long run. Numerous study designs recommend MoCA evaluation past the 6 month time point^7^. Late delayed neurocognitive decline has long relied on neurocognitive assessments, and there is no gold standard in the detection of potential late delayed neurocognitive decline early on. Several studies have suggested methods to detect markers of late delayed neurocognitive decline^8^. T1- and T2-weighted magnetic resonance (MR) imaging lesion necrosis is not consistently detectable in cases of late delayed neurocognitive decline in early stage^9^. The effect of late delayed neurocognitive decline on quality of life has become increasingly important as the effectiveness of treatment has led to recovery and a longer life span of patients. It is highly interesting to detect potential radiation-induced microstructural alterations early on which can inform the course of treatment to prevent long lasting side effects.

Diffusion related technology has been shown to be more sensitive than T1-and T2-weighted MR imaging for detecting subtle brain damage. There is evidence that Diffusion tensor imaging (DTI) is superior to traditional imaging modalities in the detection of radiation-induced temporal white matter damage 10. DTI relies on a diffusion model that assumes a Gaussian distribution of movement of water molecules. However, tissue material heterogeneity presenting as diffusive barriers such as cell membranes and organelles can invalidate the Gaussian model. Diffusional kurtosis imaging (DKI) characterizes the degree to which diffusion deviates from Gaussian behavior^11^. DKI-derived kurtosis and diffusion metrics are powerful indicators of microstructural information in the brain. DKI has been successfully used to evaluate aging, attention deficit hyperactivity disorder, cerebral glioma, epilepsy, and head and neck cancer in vivo, and has been shown to capture diffusion and kurtosis alterations especially well in white matter^12^, ^13^.

The aim of this study is to evaluate whether DKI can detect temporal microstructure change associated with late delayed neurocognitive decline not visible by traditional MRI modalities in the early stage post-IMRT in NPC patients.

## Patients and Methods

### Patients

This study was approved by the Ethics Committee of Hainan Gerenal Hospital. Written informed consent was obtained from all subjects. Fifty-four NPC patients (mean age, 48.74±12.85 years, range 20-71 years, 15 female) undergoing IMRT were enrolled in this study.

**Figure 1:**
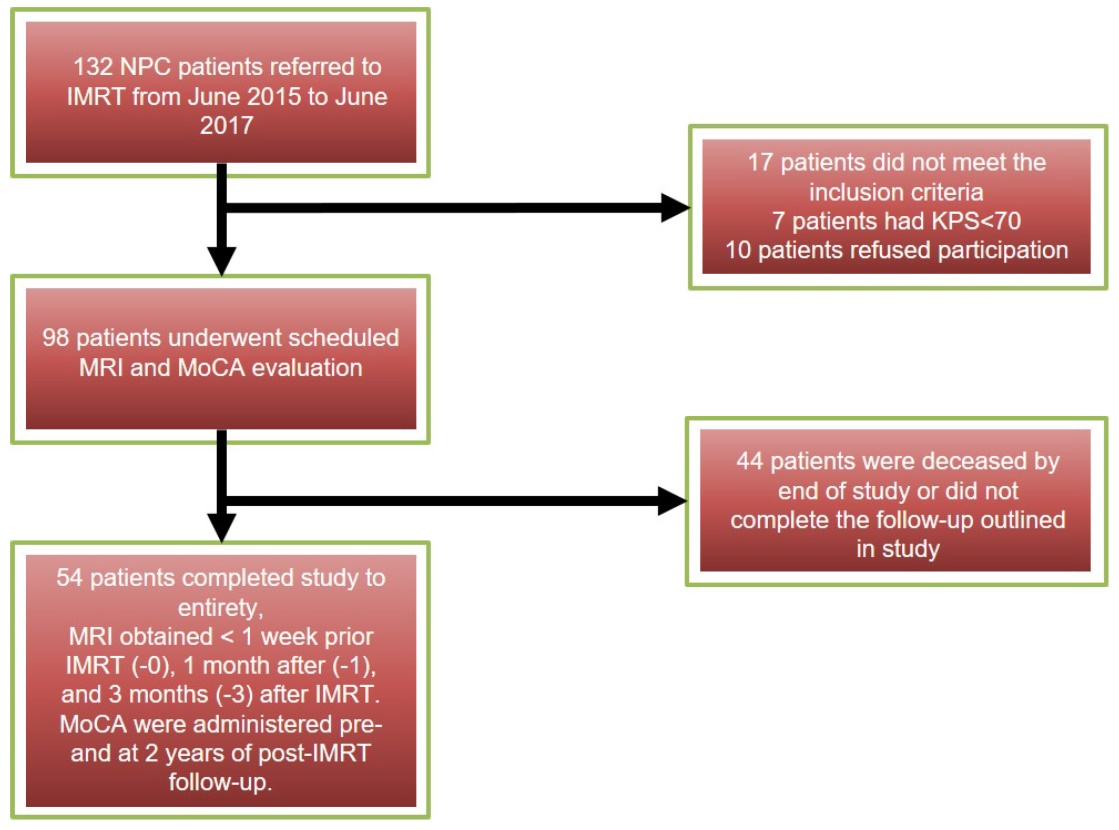
The workflow of the patients included and the study criteria.

All NPC patients were confirmed pathologically, with staging from T1N0M0 to T4N3M0 (American Joint Committee on Cancer (AJCC) Cancer Staging Manual, Eighth Edition, 2017)^14^. Patients met inclusion criteria by (1) having Karnofsky performance status (KPS) ≥ 70 %, (2) undergoing each MRI follow-up in our hospital, (3) having no evidence of tumor invading temporal lobes or temporal abnormal performance on conventional MR images. Patients were excluded who had (1) MR imaging (eg, artificial cochlea, cardiac pacemaker implantation) or radiotherapy contraindications, (2) suffered from other neurological or psychiatric diseases, (3) had been treated by radiotherapy previously, (4) had brain metastases or trauma, (5) a baseline Montreal Neurocognitive Assessment (MoCA) score <25. All subject MRIs were obtained at 3-time points: < 1 week prior IMRT (-0), 1 month after (-1), and 3 months (-3) after IMRT. Neurocognitive function tests (MoCA) were administered pre- and at 2 years of post-IMRT follow-up. The inclusion process of all the patients showed on Figure 1. All the patients were divided into neurocognitive function decline group (NFD) and neurocognitive function non-decline group (NFND) according to whether the MoCA score declined by ≥3 at the 2 years post IMRT time point. This study is distinguished from previous studies because patient data is grouped as NFD and NFND, allowing the marker to be directly linked to neurocognitive decline. This study further relates later neurocognitive decline with radiation dose.

### Radiotherapy Protocol

IMRT planning was performed on the Eclipse Treatment Planning System (Major build version 11.0.47, USA) using the simultaneous integrated boost technique. The contouring of gross tumor volume (GTV), clinical target volume (CTV), and organs at risk (OARs) complied with the International Commission on Radiation Units and Measurements (ICRU, Report 91)^15^. Target volumes were delineated slice-by-slice on treatment planning CT scans using an individualized delineation protocol, MR images for reference. A 3-mm margin was used to generate the corresponding planning target volume and planning organs at risk volume (PTV/PRV) with necessary modifications. The prescribed dose was 68.2-72.6 Gy in 31-33 fractions at 2.15-2.36Gy/fraction for the gross tumor volume of the primary tumor (GTV-P), 60-70 Gy for the gross tumor volume of cervical lymph nodes metastases (GTV-N), 60-65 Gy for CTV l (i.e., high-risk regions), and 54-58 Gy for CTV 2 (i.e., low-risk regions) and CTV-N (i.e., cervical nodal regions). OARs (brain stem, spinal cord, temporal lobe, eyes, lens, optic nerves, chiasm, salivary glands, temporomandibular joint, mandible, etc.) were contoured using three-dimensional anatomical boundaries from CT-MR fusion images. The whole organs should be outlined, including those in CTV, but not GTV. According to the Radiation Therapy Oncology Group (RTOG, 0225)^16^, the dose for at risk organs is set as a maximum by region as follows - brain stem B54 Gy, spinal cord B45 Gy, temporal lobe B54-60 Gy, optic nerve and chiasm B54 Gy, lens B9 Gy, D50 of parotid gland B30-35 Gy, temporomandibular joint B50 Gy, and mandible B60 Gy. According to the complexity and length of the individual treatment target volume, five to seven 270° (from 225° to 135°, IEC conventions) arcs were used to treat the nasopharynx and upper neck. The treatment couch was moved between arcs at 2 cm intervals craniocaudally. The mean dose (volume-weighted average dose) of the two sides temporal lobe was obtained from the co-registered radiation dose map on the planning system. In all patients, 36 patients received concurrent chemotherapy, and 15 received adjuvant chemotherapy. The concurrent chemotherapy regimen consisted of five to six cycles of injection cisplatin (CDDP; 30-40 mg/m^2^) weekly. Among the 15 patients receiving three to four cycles of adjuvant chemotherapy, eleven received CDDP (75 mg/m^2^) on day 1 and 5-fluorouracil (5-FU; 500 mg/m^2^) on days 1-5, and the remaining four patients were treated with CDDP and docetaxel (both dosed at 75 mg/m^2^) on day 1, and these cycles were repeated every 3 weeks.

### MRI Protocol

All MR imaging was performed using a 3-T MR system (Skyro, Siemens Healthcare, Erlangen, Germany) with a 20-channel head/neck coil. The routine MRI protocol included T1-weighted and proton density (Pd)-weighted images for every subject. DKI employed a single-shot echo-planar imaging sequence and array spatial sensitivity encoding technique with the following imaging parameters: repetition time (TR) 7700 ms, echo time (TE) 88 ms, the field of view (FOV) 220*220 mm2, voxel size 2.5*2.5*4 mm2, slice thickness 4 mm. Images were collected along 30 diffusion gradient encoding directions with b values of 10, 500, 1000, and 1,500 sec/mm2. The following measures are taken to ensure image quality - The patient's head is fixed in all directions before scanning and instruct patients to rest during scanning. DKI acquisition is performed first to ensure image quality. DKI and other sequences use the same location line. Before post-processing, DKI and PDWI are co-registered. PDWI was used to outline the ROI, and DKI was used to derive quantitative values. The coregistered outline is used in DKI to extract the ROI specific parameter values.

### DKI analysis

**Figure 2:**
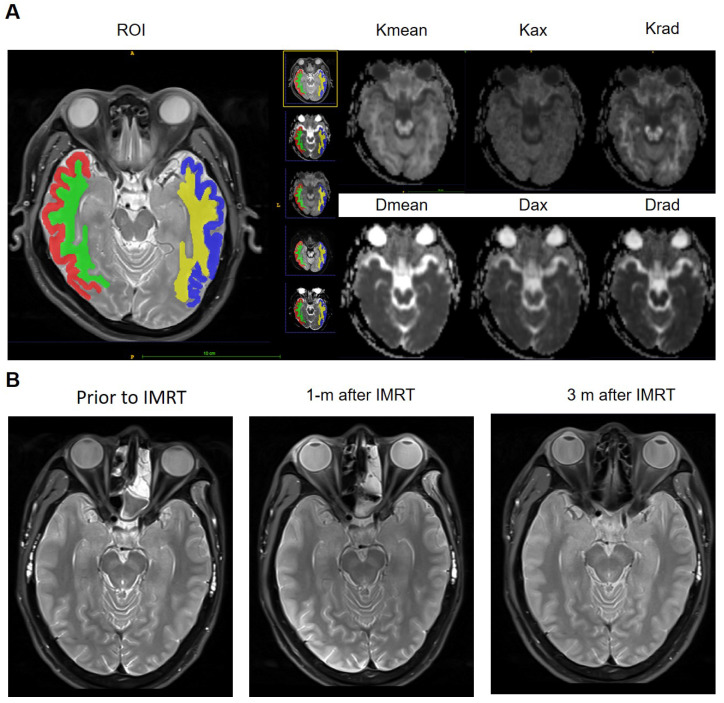
A, ROIs of temporal white matter and gray matter were delineated on the axial image of the hippocampal / hippocampus body largest level, Pd-weighted imaging as an automatic reference, and DKI metrics mappings (Kmean, Kax, Krad, Dmean, Dax, and Drad) for quantitative assessment. B, A 30-year old male NPC patient present neurocognitive function decline 2 years after IMRT. However, Pd-weighted images at the three-time points, before treatment, 1 month, and 3 months after IMRT, all did not show any abnormal signal intensity in the bilateral temporal lobe.

Post-processing of DKI raw data was performed using the Diffusional Kurtosis Estimator (DKE, version 2.6, built on February 25, 2015) software. The Kurtosis (Kmean, Kax, and Krad) and Diffusivity (Dmean, Dax, and Drad) metrics were measured using the ITK-SNAP (version 3.4.0, http://www.itksnap.org) software^17^. The region of interest (ROIs) were delineated on the axial image of the hippocampal / hippocampus body largest level (temporal gray and white matter on both sides) on the DKI maps and Pd-weighted imaging (automatic synchronized three-dimensional registration performed on the ITK-SNAP software for visualization of data). This segmentation is performed by a radiologist (R.L., with 3 years' experience) and confirmed by a senior radiologist (W.H., with 11 years' experience). The two sides of the middle cerebral artery, lateral ventricles, and other obvious artifacts are excluded manually (Figure 2 A).

### Neurocognitive function tests

The Montreal Cognitive Assessment (MoCA, Beijing version) was used to assess neurocognitive function prior- and 2-year after radiotherapy in NPC patients, including eight cognitive domains: visuospatial/executive, naming, registration, attention, language, abstraction, delayed recall, and orientation. The time to administer the evaluation is approximately 10 min. As a comprehensive measure including several cognitive tasks, the MoCA total score (range: 0-30) reflects global cognitive performance. MoCA scores that decreased ≥3 after IMRT were considered as neurocognitive decline^18^. Patients with <12y of education and age>65 receive a bonus point for corrections^19^. We used a score of <26 to define cognitive impairment.

### Statistical analysis

Statistical analysis was performed with Statistical Package for Social Sciences (SPSS Mac 20.0). All the presented data are expressed as mean±standard deviation. A Mann-Whitney *U* test was used to evaluate differences of the demographic, treatment protocol, radiation dose, and DKI indices between two groups. Receiver operating characteristic (ROC) analysis was used to evaluate the predictive performance for a late delayed neurocognitive decline. The results of statistical tests were considered statistically significant at *p* < 0.05.

## Results

### Delayed neurocognitive decline

None of the participants had neurocognitive impairment at baseline, while 19 (35.19%) participants exhibited neurocognitive impairment at 2 years after radiotherapy with MoCA<26 as a cutoff. 16 (29.63%) participants exhibited delayed neurocognitive decline (MoCA declined ≥3) belonging to the NFD group, with the other 38 (70.37%) participants belonging to the NFND group. The overlap between these groups is N= 15 patients.

### DKI of Temporal Lobe Indicate Neurocognitive Decline

**Figure 3:**
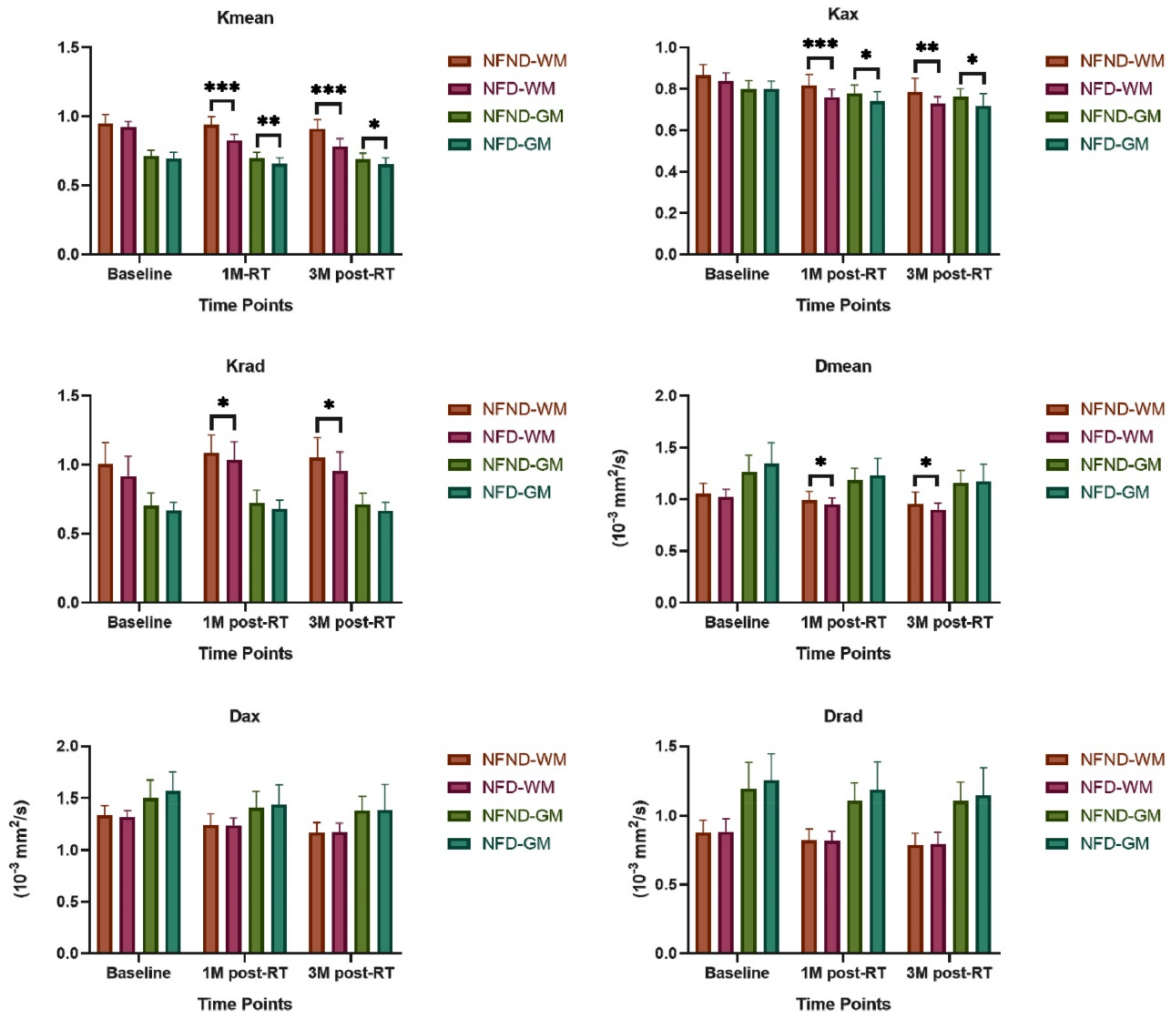
DKI characteristics of the late delayed neurocognitive decline. Kurtosis is more sensitive than Diffusivity metrics in differentiating the NFD from the NFND group. Abbreviation: NFND, neurocognitive function non-decline; NFD, neurocognitive function decline; 1M, 1 month; 3M, 3 months; WM, white matter; GM, gray matter. *(*p*<0.05), **(*p*<0.01), and ***(*p*<0.001) indicates statistical significance.

There was no significant difference in age (*p*=0.267), gender ratio (*p*=0.77), education (*p*=0.415) and T stage (*p*=0.405) between NFD and NFND group (Table 1). The baseline of both Kurtosis (Kmean-0, Kax-0, and Krad-0) and Diffusivity (Dmean-0, Dax-0, and Drad-0) does not show any significant difference between the two groups (*p*>0.05). Kurtosis (Kmean-1, Kmean-3, Kax-1, Kax-3, Krad-1, and Krad-3) and Diffusivity (Dmean-1 and Dmean-3) of white matter were significantly lower in the NFD group than NFND (*p*<0.05). Axial Kurtosis (Kax-1, Kax-3) of gray matter was significantly lower was in the NFD group than NFND (*p* < 0.05) (Table 2; Figure 3). Pd-weighted images did not show any abnormal signal intensity changes in the bilateral analysis of the temporal lobe (Figure 2B).

**Figure 4:**
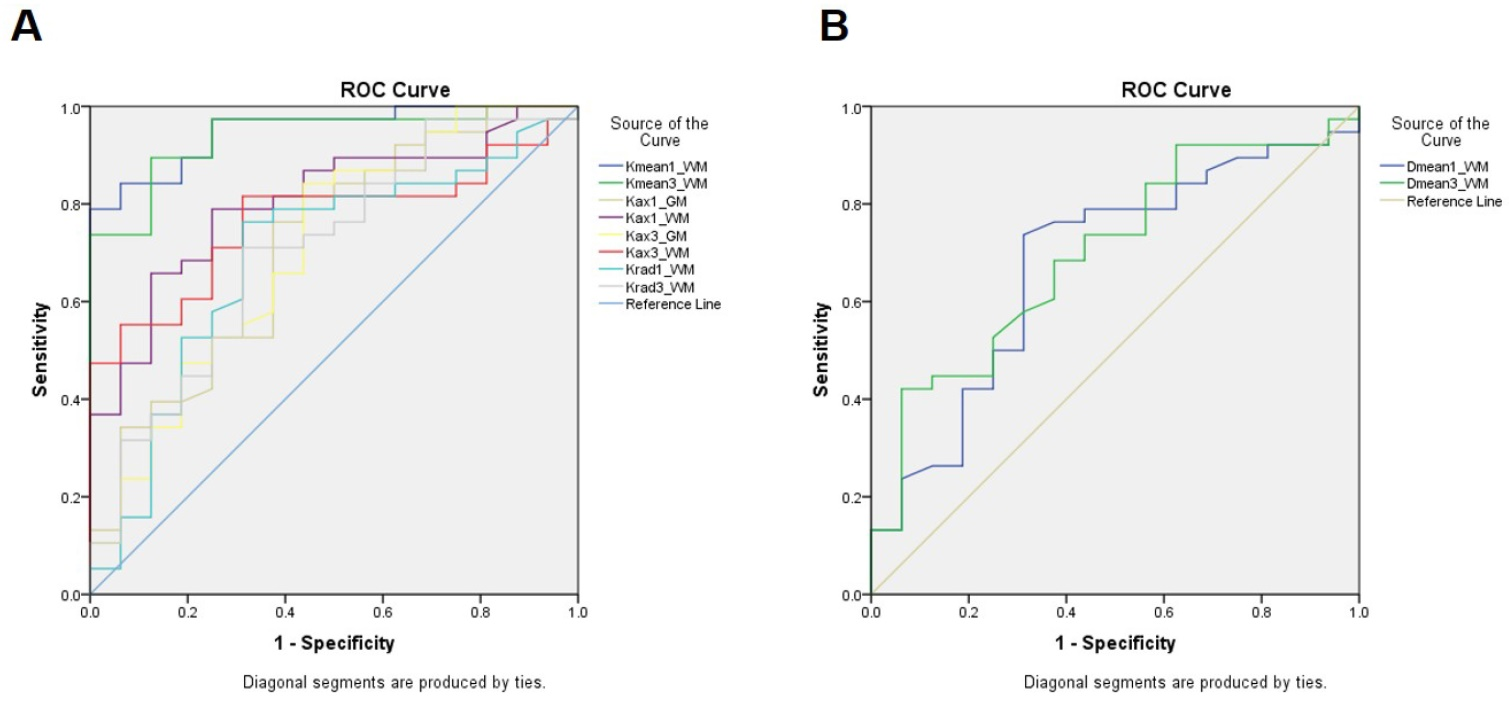
The ROC of all the parameters that have a significant difference between the NFD and the NFND group. A, The ROC curves of Kurtosis parameters; B, the ROC curve of Diffusivity parameters.

The ROC of all the parameters that have a significant difference between the groups is displayed in Figure 4. Among them, Kmean-1 of white matter performed best in predicting neurocognitive impairment through change in MoCA score after radiotherapy (area under the curve (AUC) =0.951, cutoff=0.878, sensitive=84.2%, specificity=87.5%), indicating an early change in Kmean could predict late delayed neurocognitive decline.

### Dose Dependence

**Figure 5:**
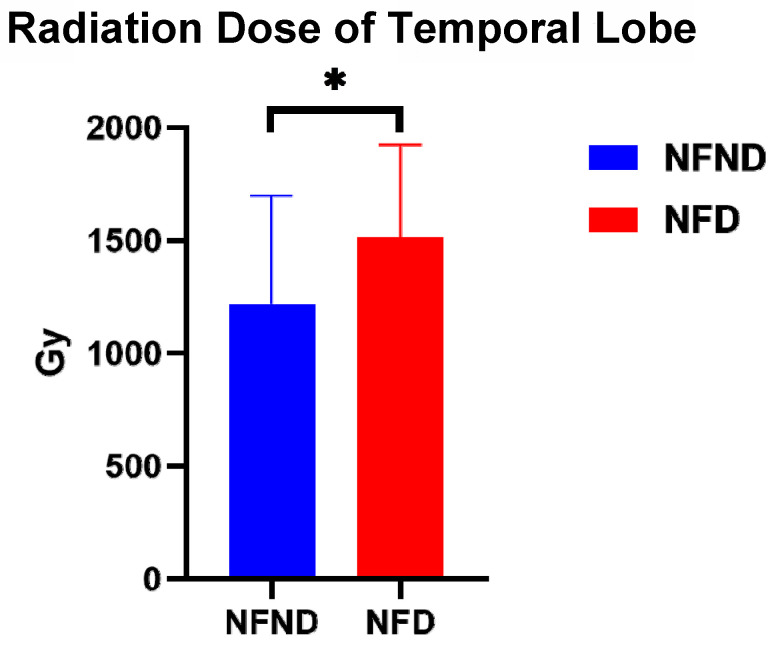
The temporal lobe dose of NFD (1511.85±411.06) is significantly higher than that of the NFND group (1217.91±480.4). Abbreviation: NFND, neurocognitive function non-decline; NFD, neurocognitive function decline; WM, white matter. *(*p*<0.05), **(*p*<0.01), and ***(*p*<0.001) indicates statistical significance.

When comparing the radiation dose of the two groups, NFD and NFND, it is found that the radiation dose of NFD (1511.85±411.06) is significantly higher than that of the NFND group (1217.91±480.4) (*p*=0.031) (Figure 5).

## Discussion

In this study, we find that Kurtosis and Diffusivity metrics derived from DKI can distinguish delayed neurocognitive decline at the acute and early delayed radiation phase in otherwise normally appearing temporal lobe tissue when evaluated by conventional MRI as seen in Figure 2B. Kmean-1 of white matter performed best in predicting radiation-induced late delayed neurocognitive decline compared to other Kurtosis and Diffusivity metrics. These findings are consistent with our hypothesis that early microstructural injury of the temporal lobe, especially white matter degradation, has a direct causative relation to late delayed neurocognitive decline. They also support our proposal that by identifying patients on a steeper and quicker “trajectory” of microstructure injury to the temporal lobe, one may be able to predict which patients are at risk for radiation-induced neurocognitive decline.

The temporal lobes of radiation treated NPC patients are at risk because they lie in the path of the radiation beams, particularly the lower-medial aspects of the lobe^20^. J. McDowell et al.^21^ reported that 32% of NPC patients scored as neurocognitively impaired with a MoCA cut-off of 23, while 70% with a cut-off of 26, and with a median follow-up of 7.5 years. Our results showed 29.63% present with neurocognitive decline, and 35.19% present with neurocognitive impairment at 2 years post treatment during the follow-up evaluation. This report is lower than the J. McDowell report because we only included participants without temporal lobe abnormality on conventional MR. Another possible reason was our relatively short follow-up. We choose the cut-off of 26 for the MoCA score in order to included mild cognitive impairment more sensitively and provide information that is more sensitive to baseline patient variability. In the neurocognitive decline classification of patients, the MoCA score post-treatment is compared to the score pre-treatment. While clinical practice often defines the cutoff for neurocognitive impairment as a MoCA score < 26 without pretreatment comparison, comparing to the patient's baseline provides a more robust measure of neurocognitive decline. This allows for an increased credibility in the results. It is probable that the clinical recommendation for a cutoff score comes from the potential that not all studies and clinical practices will have a pretreatment reading, and a score of 26 as a cutoff will allow for a single seating evaluation. With a pretreatment score available as in this study, it is more robust to use the patient specific baseline as a comparison.

A series of pathological changes of the temporal lobe after receiving radiation is the basic mechanism of neurocognitive impairment. In the acute and early delayed radiation phase, cell degeneration, edema, and blood-brain barrier injury play a major role in the pathogenesis of irradiated brain, which could usually be cured after active treatment. Injuries to blood vessels, myelin, and glial cells play a major role in the later delayed radiation phase, which could cause temporal lobe necrosis in severe cases^22^. Temporal lobe necrosis has been historically regarded as a marker of neurocognitive impairment. However, temporal lobe necrosis means that injury is already irreversible and intervention will be less efficacious. Published studies use imaging technology to detect early radiation-induced injury of the normal-appearing temporal lobe associated with cognitive changes^8^, ^23^. Chapman et al.^8^ demonstrated that post-radiotherapy changes in verbal recall scores were linearly correlated with late changes in cingulum diffusivity (λ||) derived from diffusion tensor imaging. λ|| detects the diffusion change along the axon and is more sensitive to white matter edema or injury. DTI has a less accurate underlying pretense that water molecules within the brain follow a perfectly Gaussian diffusion behavior. DKI incorporates the non-Gaussian diffusion behavior and provides more tissue sensitive information for analysis. MK/Kmean derived from DKI has been used to detect the temporal lobe necrosis in early stage^11^. We used DKI of 30 directions to determine whether DKI can distinguish patients at risk of neurocognitive impairment at the late delayed radiation phase. We found Kurtosis more sensitive than Diffusivity for differentiating NFD from the NFND group. Consistent with DKI theory, Kurtosis is more sensitive to microstructural change and organizational complexity^24^. This is probably due to local inflammation and neural degradation as a result of radiation nonspecificity leading to an increase in tissue heterogeneity which is readily detected by Kurtosis more so than Diffusivity given the underlying assumptions in each model. Among the three Kurtosis metrics, Kmean and Kax tend to be better in predicting late delayed neurocognitive impairment. A cross-sectional study also showed that grey and white matter MK values were significantly lower at 6 months and 1 year after radiotherapy in patients with NPC^25^,^7^. Kmean/MK, the average apparent kurtosis along all diffusion-encoding directions, has been shown in previous studies to be a marker of both pathological and developmental changes in neural tissue^26^. Kax should be the most sensitive indicator in detecting white matter axon damage as it is the kurtosis along the axial direction of the diffusion ellipsoid^27^. As such, Kax is more sensitive to direction-dependent tissue complexity, and would be more effective if it is used to detect the destruction of white matter cellular injury^24^. It is also notable that λ|| tends to be larger in magnitude than λ⊥. However, λ⊥ can be sensitive to changes in myelination. Krad represents the radial direction of the diffusion ellipsoid^27^. As such, Krad may indicate changes in myelination due to radiation-induced damage. Our results showed the AUC of Kmean in predicting neurocognitive decline was slightly larger than that of Kax in ROC. The possible reason is Kmean has the ability to detect anisotropic properties of diffusion. Furthermore, radiation induced demyelination will also influence Kmean more than Kax. This supports the possibility that Kmean might become a sensitive early-stage biomarker for water diffusion restriction caused by various temporal white matter microstructure changes. Krad showed less ability to identify the late delayed neurocognitive than Kax and Kmean. This supports the theory that axonal damage is a large underlying feature of microstructural change that leads to the eventual potential neurocognitive decline.

Previous DTI studies only show temporal white matter changes after radiotherapy^28^, ^29^. Gray matter has a high neuronal cell body cell (soma) distribution and is seldom studied. In this research, Kax in gray matter was also found to be significantly different in the NFD and NFND group. Similar to Wang's study^25^, DKI could be used as a new method for the early detection of radiation-induced temporal lobe injury in gray matter in patients with NPC. The pathological basis is considered to be protein accumulation or iron deposition in addition to cellular degradation^30^. Thus, we conclude, alongside previous studies, that DKI is sensitive to detecting pathology in both the gray and white matter^31^. However, consistent with published studies^32^, white matter is still more vulnerable to radiation-induced injury than gray matter.

We found that the radiation dose was significantly higher in the NFD group than the NFND group. The demographic data, tumor stage, and chemotherapy regimens were not significantly different between the two groups at baseline. Radiation dose might one of the most important factors leading to the late delayed neurocognitive decline. The results indicate that NFD patients have temporal lobes with higher received doses of radiation than NFND patients. McDowell et al.^21^ demonstrated 32% of NPC survivors exhibited cognitive impairment after radiotherapy. However, they found no evidence of radiation dose to temporal lobe influencing cognition, despite previous work indicating this^33^. DKI, vulnerable to microstructural changes, might be more sensitive in the detection of temporal lobe changes than neurocognitive function scores like MoCA. This paper is novel by combining DKI and MoCA to demonstrate that late delayed neurocognitive decline is dose-dependent.

Chemotherapy is a viable risk factor for cognitive dysfunction. In our protocol, all patients receive different chemotherapy regimens. Guo et al.^6^ found no brain microstructural or MoCA changes in the subcohort of NPC patients with induction chemotherapy before the initiation of RT. Qin et al.^34^ domenstrated that NPC patients who receive many cycles of concurrent chemotherapy may be at an increased risk of depression after completion of IMRT.

There are some additional limitations in our study. First, the MoCA is an incomplete cognitive screening tool that is not sensitive to executive functions, verbal and visual memory, and attention, amongst other factors. These other factors have also been shown as impaired through assessment in previous studies^35^. A comprehensive cognitive evaluation could be performed as a part of future research to elucidate extent of cognitive impairment. Second, most of the participants enrolled in the present study were treated with concurrent chemotherapy/adjuvant chemotherapy and radiotherapy. Although we found that the chemotherapy regiment was not markedly different between the decline and no decline groups, a future study should enroll chemotherapy only and radiotherapy only groups to elucidate treatment protocol effects. It will also be insightful to study similar markers in NFD patients and NFND patients who receive the same dose of radiation. While NFD patients are more likely to have received a higher dose of radiation, other underlying genetic and physiological mechanisms modulate sensitivity to radiation. In understanding whether the same DKI markers serve as useful metrics to evaluate neurocognitive impairment in patients with the same dose but differing outcomes will have utility in the practical application of these techniques in the clinic. Furthermore, late delayed cognitive decline is detected by early markers given the underlying assumption that the initial radiation assault causes microstructural changes early on that correlate with late delayed neurocognitive decline. However, it is unclear which particular changes are being detected and whether these changes are more correlated with recoverable acute injuries (inflammation, mild vascular assault) or nonrecoverable injuries (substantial cellular death, severe demyelination). Being able to distinguish what is exactly being detected by Kmean and other DKI metrics will allow for a more informed understanding of how effective this method is as an early marker of neurocognitive decline. This can be accomplished by studying ex vivo tissue models or tissue phantoms that model each process and allow for better understanding of what microstructural changes DKI is most sensitive to.

## Conclusion

DKI is a potent method for the early diagnosis of neurocognitive impairment and radiation-induced temporal lobe microstructural injury. Kurtosis is more sensitive to detecting microstructural changes in tissue than Diffusivity. The Kmean early after radiotherapy has the best prediction performance for a late delayed neurocognitive decline. Furthermore, late delayed neurocognitive decline shows a dose-dependency. This research provides insight for future clinical practice to use early stage DKI markers to adjust treatment prior to irreversible radiation induced damage for the best patient outcome.

## Figures and Tables

**Table 1:** Patient characteristics (N=54)

Characteristic	No. of patients	Years	%
Age			
Mean±SD		48.74±12.851	
Range		20~71	
Gender			
Male	39		72.22%
Female	15		27.78%
Education level			
<6y	1		1.86%
6-12y	40		74.07%
>12y	13		24.07%
T Stage (7^th^ UICC)			
T1-2	29		53.7%
T3-4	25		46.3%
Treatment protocol			
RT	3		5.56%
Concurrent Chemoradiotherapy	36		66.67%
Adjuvant Chemotherapy+RT	9		16.67%
Adjuvant+Concurretn Chemotherapy+RT	6		11.1%

SD, standard deviation; RT, radiotherapy;

**Table 2:** DKI characteristics of NPC patients received IMRT

Parameter	NFD Group (N=16)		NFND Group (N=38)		*P* value	
	WM	GM	WM	GM	WM	GM
Kmean-0	0.92±0.04	0.69±0.05	0.95±0.07	0.71±0.04	0.103	0.293
Kmean-1	0.83±0.04	0.66±0.04	0.94±0.06	0.69±0.04	0.000	0.009
Kmean-3	0.78±0.06	0.65±0.05	0.91±0.07	0.69±0.05	0.000	0.044
Kax-0	0.84±0.04	0.8±0.04	0.87±0.05	0.8±0.04	0.096	0.649
Kax-1	0.76±0.04	0.74±0.05	0.82±0.05	0.78±0.04	0.000	0.013
Kax-3	0.73±0.03	0.72±0.06	0.78±0.07	0.76±0.04	0.002	0.017
Krad-0	0.92±0.15	0.67±0.06	1.00±0.16	0.70±0.09	0.066	0.191
Krad-1	1.02±0.15	0.68±0.06	1.11±0.15	0.72±0.09	0.028	0.088
Krad-3	0.96±0.14	0.67±0.06	1.05±0.14	0.71±0.08	0.019	0.099
Dmean-0	1.02±0.08	1.35±0.2	1.05±0.1	1.26±0.16	0.348	0.120
Dmean-1	0.95±0.06	1.23±0.16	0.99±0.09	1.18±0.12	0.040	0.460
Dmean-3	0.90±0.07	1.17±0.17	0.95±0.11	1.15±0.12	0.022	0.962
Dax-0	1.32±0.07	1.57±0.18	1.34±0.10	1.50±0.18	0.719	0.225
Dax-1	1.24±0.08	1.44±0.19	1.24±0.11	1.41±0.16	0.544	0.733
Dax-3	1.17±0.09	1.39±0.24	1.17±0.10	1.38±0.13	0.769	0.389
Drad-0	0.88±0.10	1.25±0.19	0.88±0.10	1.19±0.19	0.842	0.101
Drad-1	0.82±0.07	1.18±0.20	0.82±0.08	1.11±0.13	0.925	0.150
Drad-3	0.79±0.09	1.14±0.20	0.79±0.09	1.10±0.14	0.733	0.489
Radiation dose	1511.85±411.06		1217.91±480.40		0.031	
